# KCa3.1/IK1 Channel Regulation by cGMP-Dependent Protein Kinase (PKG) via Reactive Oxygen Species and CaMKII in Microglia: An Immune Modulating Feedback System?

**DOI:** 10.3389/fimmu.2015.00153

**Published:** 2015-04-08

**Authors:** Roger Ferreira, Raymond Wong, Lyanne C. Schlichter

**Affiliations:** ^1^Genetics and Development Division, Toronto Western Research Institute, University Health Network, Toronto, ON, Canada; ^2^Department of Physiology, University of Toronto, Toronto, ON, Canada

**Keywords:** KCa3.1/KCNN4/IK1/SK4 regulation, cGMP-PKG signaling, reactive oxygen species signaling, Ca^2+^-CaM-CaMKII signaling, patch-clamp electrophysiology, fura-2 calcium measurement, alternative-activated microglia, interleukin-4 stimulation

## Abstract

The intermediate conductance Ca^2+^-activated K^+^ channel, KCa3.1 (IK1/SK4/*KCNN4*) is widely expressed in the innate and adaptive immune system. KCa3.1 contributes to proliferation of activated T lymphocytes, and in CNS-resident microglia, it contributes to Ca^2+^ signaling, migration, and production of pro-inflammatory mediators (e.g., reactive oxygen species, ROS). KCa3.1 is under investigation as a therapeutic target for CNS disorders that involve microglial activation and T cells. However, KCa3.1 is post-translationally regulated, and this will determine when and how much it can contribute to cell functions. We previously found that KCa3.1 trafficking and gating require calmodulin (CaM) binding, and this is inhibited by cAMP kinase (PKA) acting at a single phosphorylation site. The same site is potentially phosphorylated by cGMP kinase (PKG), and in some cells, PKG can increase Ca^2+^, CaM activation, and ROS. Here, we addressed KCa3.1 regulation through PKG-dependent pathways in primary rat microglia and the MLS-9 microglia cell line, using perforated-patch recordings to preserve intracellular signaling. Elevating cGMP increased both the KCa3.1 current and intracellular ROS production, and both were prevented by the selective PKG inhibitor, KT5823. The cGMP/PKG-evoked increase in KCa3.1 current in intact MLS-9 microglia was mediated by ROS, mimicked by applying hydrogen peroxide (H_2_O_2_), inhibited by a ROS scavenger (MGP), and prevented by a selective CaMKII inhibitor (mAIP). Similar results were seen in alternative-activated primary rat microglia; their KCa3.1 current required PKG, ROS, and CaMKII, and they had increased ROS production that required KCa3.1 activity. The increase in current apparently did not result from direct effects on the channel open probability (*P*_o_) or Ca^2+^ dependence because, in inside-out patches from transfected HEK293 cells, single-channel activity was not affected by cGMP, PKG, H_2_O_2_ at normal or elevated intracellular Ca^2+^. The regulation pathway we have identified in intact microglia and MLS-9 cells is expected to have broad implications because KCa3.1 plays important roles in numerous cells and tissues.

## Introduction

Following the discovery of a Ca^2+^-dependent K^+^ efflux (“Gardos” channel) in red blood cells (1), early patch-clamp studies focused on the intermediate-conductance Ca^2+^-dependent K^+^ (IK) channel in thymic T cells, B lymphocytes (2), and T cells (3, 4). After the *KCNN4* gene was cloned (5–7) and identified as the IK channel (also called KCa3.1, IK1, IKCa1, SK4); its upregulation in activated T lymphocytes and crucial role in their proliferation (8, 9) generated interest in targeting this channel for immunosuppression [reviewed in Ref. (10–12)]. Initially, the KCa3.1 channel was thought to be absent from the CNS, but then *KCNN4* transcripts and KCa3.1 protein were detected in microglia (13, 14), astrocytes, and some neurons (15–17). Many CNS disorders involve inflammation and microglial activation, and KCa3.1 blockers or knockdown have improved the outcome in animal models of trauma, spinal cord injury, ischemic stroke, multiple sclerosis, and Alzheimer’s disease [reviewed in Ref. (18, 19)]. Thus, there is increasing interest in roles and regulation of KCa3.1 in the CNS.

KCa3.1 expression, activity, and contributions to T cell functions are governed by the cells’ activation state but until recently, little was known about this aspect for microglia. In rat microglia, we found that KCa3.1 contributes to production of reactive oxygen species (ROS) (14), to p38 MAPK activation, nitric oxide, and peroxynitrite production in classical-activated microglia (“M1,” by analogy with macrophages polarized by T cells), and to their capacity to kill neurons *in vitro* and *in vivo* (15). We recently discovered that when rat microglia are skewed to the anti-inflammatory “alternative” (M2) activation state using interleukin-4, both KCa3.1 expression and current are highly upregulated through the type I IL-4 receptor and subsequent signaling through JAK3, Ras/MEK/ERK, and the transcription factor, AP-1 (20). While KCa3.1 is involved in microglial ROS production, it is not known if its activity is regulated by ROS. There is some indirect evidence that this might occur. ROS can evoke Ca^2+^ release from internal stores in Jurkat T cells (21) and pancreatic β-cells (22). We found that, in microglia, the KCa3.1 current is functionally coupled to Ca^2+^-release activated Ca^2+^ (CRAC) channels. When CRAC was activated through P_2_Y_2_ metabotropic purinergic receptors, this activated KCa3.1 channels, and they then contributed to the microglia migratory phenotype (23). Migration is also increased in alternative-activated microglia, and this depends on KCa3.1 (20, 24).

KCa3.1 activity is also post-translationally regulated. Most fundamental is its absolute requirement for Ca^2+^ and calmodulin (CaM) in order for the channels to open (8, 25) and to traffic to the cell surface (26). CaM is bound to the C terminus of the channel but there is evidence that this interaction can be modulated. We found that the KCa3.1 current is inhibited by cAMP kinase (PKA) through a single phosphorylation consensus site (S334 in human; S332 in rodent, which is two amino acids shorter), and a consequent decrease in CaM binding to the channel (27). Many years ago, we observed that the KCa3.1 current in human T lymphoblasts was reduced by the CaM kinase inhibitor, KN-62, but only at 37°C (not room temperature) and not in KCa3.1 heterologously expressed in CHO cells (8). Both observations suggested an indirect modulation by CaM kinase, but this was not investigated further.

More recent studies suggested a possible link between KCa3.1 and CaMKII, ROS and cGMP-protein kinase (PKG). In cardiac and neuronal cells, the PKG pathway can stimulate ROS production and activate CaM/CaMKII signaling (28–30). Numerous stimuli can activate PKG in immune cells, and we noticed that the Ser334/S332 site in KCa3.1 that is regulated by PKA (27) is also a potential consensus site for phosphorylation by PKG. Thus, the present study was designed to test whether KCa3.1 is directly inhibited by cGMP/PKG, and if not, whether it is indirectly regulated and whether this involves crosstalk between Ca^2+^, ROS, and CaM/CaMKII. First, we analyzed the endogenous KCa3.1 current in a rat microglial cell line because we found that every MLS-9 cell expresses a robust KCa3.1 current that can be easily isolated (23, 31). Then, we corroborated the salient findings on native KCa3.1 channels in primary rat microglia. Importantly, regulation of native channels was studied using the perforated-patch recording configuration to maintain intracellular soluble mediators and biochemical signaling pathways, and to allow cytoplasmic Ca^2+^ to remain at physiological levels and change with treatments.

## Materials and Methods

### Cells

Primary cultured rat microglia and the MLS-9 microglia cell line were used to study native KCa3.1 channels, and transfected HEK293 cells were used to facilitate single-channel analysis.

#### Primary rat microglia

Microglia were isolated from brains of 1–2-day-old Sprague-Dawley rats of either sex (Charles River, St. Constant, QC, Canada) according to our standard protocols (15, 24, 31). The brains were harvested, meninges removed, remaining tissue minced in cold minimum essential medium (MEM; Invitrogen, Burlington, ON, Canada), and then centrifuged (300 *g*, 10 min) and re-suspended in MEM with 10% fetal bovine serum (FBS; Wisent, St. Bruno, QC, Canada) and 0.05 mg/mL gentamycin (Invitrogen). The culture medium was replaced after 2 days to remove non-adherent cells and debris. After six more days, the mixed cell cultures were shaken on an orbital shaker (65 rpm, 4–5 h, 37°C, 5% CO_2_), and then the microglia-containing supernatant was centrifuged (300 *g*, 10 min), and microglial cells were re-suspended in MEM with 2% FBS. Microglia were plated at 7 × 10^4^ cells/coverslip for 24 h, and then exposed to 20 ng/mL rat recombinant interleukin-4 (IL-4; R&D Systems Inc., Minneapolis, MN, USA) for 1 or 2 days (37°C, 5% CO_2_) before analyzing ROS production, or for 2 days (37°C, 5% CO_2_) before analyzing KCa3.1 currents. We previously showed that IL-4 shifts them to an alternative-activated state (24, 31) and upregulates *KCNN4* mRNA and the KCa3.1 current (20, 27), whereas untreated rat microglia are non-activated (32).

#### MLS-9 cells

About 20 years ago, we derived the MLS-9 cell line by treating rat microglia cultures for several weeks with M-CSF (colony stimulating factor-1) (33). Although we do not know whether these cells reflect an alternative activation state, M-CSF can shift macrophages to an alternative-activated state (34), and it suppresses the response of microglia to lipopolysaccharide (LPS) (35). We have used MLS-9 cells extensively to study microglial K^+^, Cl^−^ and TRPM7 channels (23, 31, 36–41). After thawing, the cells were cultured (37°C, 5% CO_2_) for several days in MEM with 10% FBS and 0.05 mg/mL gentamycin. They were harvested in phosphate buffered saline (PBS) with 0.25% trypsin and 1 mM EDTA, washed with MEM, centrifuged (300 *g*, 10 min), and re-suspended in MEM. MLS-9 cells were plated on glass coverslips in 12-well plates (4.5 × 10^4^ cells/coverslip) for patch-clamping and Fura-2 analysis, or in 96-well plates (6.0 × 10^4^ cells/well) for measuring intracellular ROS.

#### Transfection of HEK293 cells

HEK293 cells (embryonic neuronal tumor from a human female kidney) were grown for several days in Dulbecco’s modified Eagle’s medium (DMEM; Invitrogen) with high glucose, 10% FBS, 100 mg/L penicillin–streptomycin (Invitrogen). They were harvested in PBS with 0.25% trypsin and 1 mM EDTA, washed with MEM, centrifuged (300 *g*, 10 min), and re-suspended in MEM. The human KCa3.1 gene (wild-type hKCa3.1) was subcloned into the expression vector, pCMV6-XL5 (OriGene, Rockville, MD). The plasmids, pCMV6-XL5-hKCa3.1 and pEF-GFP, were co-transfected using LipofectAMINE (Invitrogen) for 36 h according to the manufacturer’s protocol. HEK293 cells were plated at 5.5 × 10^4^ cells/coverslip for single-channel patch-clamp analysis.

### Patch-clamp electrophysiology

#### Perforated-patch recordings

Endogenous KCa3.1 currents were recorded at room temperature from primary cultured rat microglia and MLS-9 cells. Perforated-patch recordings were obtained by including 200 μM amphotericin B in the pipette (intracellular) solution, which contained (in mM): 100 K aspartate, 40 KCl, 1 MgCl_2_, 0.5 CaCl_2_, 1 EGTA, 2 MgATP, 10 HEPES, pH 7.2 (adjusted with KOH), 280 mOsm. Internal free Ca^2+^ was 120 nM, as calculated by WEB-MAXC Extended software (http://www.stanford.edu/~cpatton/webmaxc/webmaxcE.htm; Stanford University). The extracellular (bath) solution contained (in mM) 125 NaCl, 5 KCl, 1 MgCl_2_, 1 CaCl_2_, 5 glucose, 10 HEPES, pH 7.4 (adjusted with NaOH), and adjusted to ~300 mOsm with sucrose. The bath solution was perfused using a gravity-driven system flowing at 1.5–2 mL/min. Recording pipettes (8–12 MΩ) were pulled from thin-walled borosilicate glass (WPI, Sarasota, FL, USA) using a Narishige puller (Narishige Scientific, Setagaya-Ku, Tokyo, Japan). Recordings were made with an Axopatch 200A amplifier (Molecular Devices, Sunnyvale, CA, USA), digitized with a DigiDATA 1322A board, filtered at 5 kHz and sampled at 10 kHz. Junction potentials were reduced by using agar bridges made with bath solution, and calculated with the pCLAMP utility. After correction, all voltages are ~5 mV more negative than shown in the figures.

#### Single-channel recordings

Inside-out patches were excised from HEK293 cells that had been transfected with hKCa3.1. Recordings were made at room temperature, sampled at 5 kHz, and low-pass filtered at 1 kHz (−3 dB cut-off frequency). Pipettes were pulled from thin-walled borosilicate glass (6–8 MΩ) and filled with an extracellular solution containing (in mM) 145 KCl, 1 MgCl_2_, and 1 CaCl_2_, 5 HEPES; pH 7.4 (adjusted with KOH), adjusted to ~300 mOsm with sucrose. The bath solution contained (in mM) 145 KCl, 1 MgCl_2_, 1 CaCl_2_, 0.1 MgATP, 5 HEPES, and 5 glucose and 1.2, 1.5 or 2.8 EGTA, to yield intracellular free Ca^2+^ concentrations of 1 μM, 500 nM, and 120 nM respectively; pH 7.2 (adjusted with KOH), ~300 mOsm. *NP*_o_, the product of the apparent number of active channels in the patch (*N*) and the channel open probability (*P*_o_) was calculated in pClamp by dividing the mean total current (*I*) by the single-channel current amplitude (*i*), where *NP*_o_ = *I/i*. The single-channel current was determined from the best Gaussian fit to the single-channel event amplitude histogram, and this also indicated the apparent number of active channels in the patch. At the end of each recording, 1 μM TRAM-34 was added to block the channels and identify them as KCa3.1.

### Measuring intracellular ROS

Primary rat microglia and MLS-9 cells in 96-well tissue culture plates were incubated with 5 μM of CM-H_2_DCFDA (5-(and-6)-chloromethyl-2′,7′-dichlorodihydrofluorescein diacetate acetyl ester) at 37°C for 1 h. After this cell-permeant reagent diffuses into cells, the acetyl group is removed by intracellular esterases, and it can then be oxidized by the intracellular reactive species, hydrogen peroxide (H_2_O_2_), hydroxyl radical (OH^•^), and peroxynitrite (ONOO^–^) (42). The resulting fluorescent adduct, dichlorofluorescein (DCF), is produced in proportion to the reactive species and remains trapped inside the cell. Before plate-reader analysis, cells were washed with the same extracellular solution used for perforated-patch recordings. Intracellular DCF fluorescence was excited at 480 nm, and measured at 530 nm with a fluorescence plate reader (Victor3 1420 Multilabel Plate Counter; Perkin Elmer Inc, Waltham, MA, USA). For each treatment, the fluorescence was averaged from two separate wells of cells cultured from one animal (for primary microglia) or from one passage (for MLS-9 cells), and then multiple *n* values were obtained using cultures from different animals or cell passages. Background subtraction was performed using control wells without CM-H_2_DCFDA. The fluorescence intensity for each treatment group was normalized to the corresponding control value.

### Intracellular free Ca^2+^

The Fura-2 imaging methods were the same as recently described (23, 27, 31). In brief, cells growing on glass coverslips (~7 × 10^4^ cells/15 mm diameter coverslip) were incubated at room temperature with 3.5 μg/mL Fura-2AM (Invitrogen) for 40 min in the dark. For recording, a coverslip was mounted in a 300 μL volume perfusion chamber (Model RC-25, Warner Instruments, Hamden, CT, USA) that contained the same bath solution as for perforated-patch recording. Responses to db-cGMP and H_2_O_2_ were assessed on different batches of cells from separate coverslips. Images were acquired at room temperature using a Nikon Diaphot inverted microscope, Retiga-EX camera (Q-Imaging, Burnaby, BC, Canada), and Northern Eclipse image acquisition software (Empix Imaging, Mississauga, ON, Canada). A Lambda DG-4 Ultra High Speed Wavelength Switcher (Sutter Instruments, Novato, CA, USA) was used to alternately acquire images at 340 and 380 nm excitation wavelengths. Images were acquired every 4 s, and the excitation shutter was closed between acquisitions to prevent photobleaching. The intracellular free Ca^2+^ concentration was calculated from the standard equation (43).

### Chemicals

Working solutions were prepared just before use by diluting fresh aliquots of stock solutions of dibutyryl-cGMP (db-cGMP), cGMP, KT5823, *N*-(2-mercaptopropionyl)glycine (MPG), myristolated autocamtide-2 related inhibitory peptide for CaMKII (mAIP), TRAM-34, apamin, riluzole, and rat recombinant IL-4. Stock solutions were prepared as follows: KT5823, TRAM-34, apamin, and riluzole were prepared in DMSO and stored at –20°C; db-cGMP, cGMP, and mAIP were prepared in distilled H_2_O and stored at –20°C; MPG was prepared in distilled H_2_O and stored at 4°C; IL-4 was prepared in sterile PBS containing 0.1% BSA and stored at –20°C. H_2_O_2_ was prepared fresh daily from a 30% w/w liquid stock (9.8 M). Aliquots of the original stock of PKG Iα holoenzyme were stored at –80°C, and diluted immediately before use in the bath solution that was used for single-channel recordings. PKG Iα holoenzyme and mAIP were obtained from Calbiochem (EMD Biosciences; San Diego, CA, USA), rat recombinant IL-4 was from R&D Systems Inc. (Minneapolis, MN, USA), and all other reagents were from Sigma Aldrich (Oakville, ON, Canada).

### Statistical analysis

Data are expressed as mean ± SEM. For single and multiple comparisons to assess treatment effects on currents, either an unpaired Student’s *t*-test or 1-way ANOVA with Tukey’s *post hoc* test was used. Changes in intracellular Ca^2+^ following treatment with db-cGMP or H_2_O_2_ were analyzed using a paired Student’s *t*-test. For analysis of intracellular ROS production, a 2-way ANOVA followed by Bonferroni’s *post hoc* test was used, with stimulation (untreated, 24 h IL-4, 48 h IL-4) and inhibitors (untreated, KT5823, TRAM-34) as the two independent variables. Analyses were conducted using GraphPad Prism ver 6.01 (GraphPad Software, San Diego, CA, USA), and statistical significance was taken as *p* < 0.05.

## Results

### PKG increases the endogenous KCa3.1 current in microglia

The MLS-9 microglial cell line was used for initial experiments because these cells have a large endogenous KCa3.1 current, and lack Kv1.3, Kv1.5, and Kir2.1 currents that are expressed in primary rat microglia (33, 44, 45). They have a KCa2.3 (SK3) current (31, 41); thus, the bath always contained the blocker, 100 nM apamin. While KCa3.1 channels in many cell types can be activated by sub-micromolar intracellular free Ca^2+^ (18), we found that the *K*_d_ is nearly 8 μM in MLS-9 cells (23), and that 1 μM Ca^2+^ failed to activate the current in primary rat microglia (20, 27). We do not know why this is the case. However, the microglial current can be activated at lower Ca^2+^ (e.g., 1 μM) by the activators, riluzole, 1-EBIO, or NS309 (20, 27, 31). These activators act as positive gating modulators that increase the Ca^2+^ sensitivity of KCa2.x and KCa3.1 channels (18).

**Figure 1:**
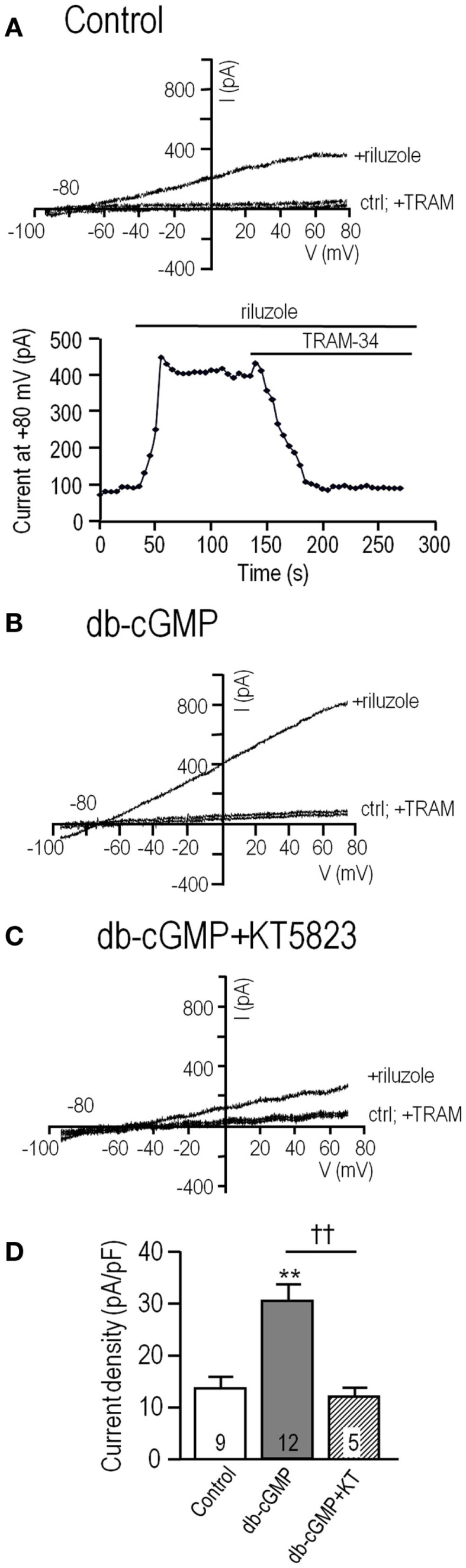
**The endogenous KCa3.1 current in MLS-9 microglial cells is increased by cGMP, which requires cGMP-protein kinase**. For all traces, the voltage protocol was a holding potential of –70 mV, and repeated ramps from –100 to +80 mV. Recordings were conducted at room temperature in the perforated-patch configuration, and riluzole was used simply to activate the KCa3.1 current at the normal low intracellular Ca^2+^ concentration. The bath always contained 100 nM apamin, a KCa2.1–2.3 channel blocker. **(A)** *Upper*: Representative current traces from a control cell (trace marked “ctrl”), followed by bath addition of 300 μM riluzole, and then 1 μM of the selective KCa3.1 blocker, TRAM-34. *Lower*: The time course of current activation and block by 1 μM TRAM-34. **(B,C)** Representative current traces from cells before and after activating the current with riluzole; with or without 1 μM TRAM-34. Cells were pre-treated with the membrane-permeant cGMP analog, db-cGMP (100 μM), for 20 min at room temperature, without **(B)** or with **(C)** 1 μM KT5823, a selective inhibitor of cGMP-protein kinase (PKG). **(D)** Summarized data from a population study using the treatments in panels A–C. For each cell, the KCa3.1 current amplitude was measured at +80 mV, as the component of the riluzole-activated current that was blocked by TRAM-34 (1 μM). The current was always normalized to the cell capacitance (in pF) to account for any differences in cell size and expressed as current density. The TRAM-34-sensitive KCa3.1 current is expressed as mean ± SEM for the number of cells indicated on each bar, and data were compared using one-way ANOVA, with Tukey’s *post hoc* test. **p < 0.01, indicates a difference from both controls and KT5823-treated cells. ††*p* < 0.01, for the comparison indicated. There was no difference between the control and KT5823-treated cells.

To study the native channels in MLS-9 and primary microglial cells, we used riluzole because it reliably activated a KCa3.1 current in perforated-patch recordings. The current was also stable enough to add TRAM-34 to confirm the channel identity and quantify the current density (20, 31). As expected, the KCa3.1 current was not activated at resting levels of intracellular Ca^2+^ in MLS-9 cells. However, a stable KCa3.1 current was activated by riluzole in all cells tested (Figure 1A). As expected for KCa3.1, current activation was independent of voltage, and it reversed close to the Nernst potential for K^+^ (–84 mV with the solutions used). The current was entirely KCa3.1 (in the presence of apamin), as demonstrated by full inhibition by the selective KCa3.1 blocker, 1 μM TRAM-34 (Figure 1A). In all subsequent experiments, the KCa3.1 current was quantified as the TRAM-34-sensitive component.

Effects of elevating intracellular cGMP were analyzed in MLS-9 cells exposed for 20 min to the membrane-permeant cyclic GMP analog, db-cGMP, before establishing a perforated-patch recording. The KCa3.1 current was much larger after db-cGMP treatment (Figure 1B) than the control current. Two observations provided the initial evidence for an indirect action of cGMP that requires intact cells (perhaps a diffusible mediator is lost when the cell integrity is disrupted). cGMP alone did not activate the channels (riluzole was required), and adding db-cGMP during whole-cell recordings did not increase the current (not shown). Therefore, we focused on perforated-patch recordings, and tested 1 μM KT5823, a membrane-permeant compound that selectively inhibits PKG (cGMP-protein kinase) with no effect on PKA (29). After treatment with db-cGMP in the presence of KT5823, only a small KCa3.1 current was activated (Figure 1C). As summarized in Figure 1D, the current density was 13.9 ± 2.1 pA/pF (*n* = 9) in control cells, more than twofold larger after db-cGMP (29.0 ± 2.6 pA/pF; *n* = 12; *p* < 0.01), and 11.3 ± 1.4 pA/pF (*n* = 5; not different from control) when the PKG inhibitor was also added. These results indicate that the current enhancement by db-cGMP required PKG.

### Lack of direct KCa3.1 activation by cGMP-protein kinase (PKG) in excised patches

The next question, whether PKG directly affects KCa3.1 channel activity, was prompted by our recent study in which KCa3.1 was assessed in excised inside-out patches from transfected HEK293 cells (27). PKA decreased the channel open probability (*P*_o_), and this was abolished by mutating the S334 site to S334A, which cannot be phosphorylated (27). Here, the rationale was that S334 is also the only putative PKG phosphorylation site, and the channel could not distinguish which kinase has phosphorylated it. We hypothesized that PKG would decrease *P*_o_ if it acts directly on the channel. For direct comparison with our earlier PKA study, we used the same experimental system: HEK293 cells transfected with human *KCNN4* (hKCa3.1). This system offered several advantages. HEK293 cells lack endogenous KCa3.1 current but after transfection, there were more active channels than in microglia (larger whole-cell currents), which made it easier to find channels in a patch, and the current was readily activated by 1 μM Ca^2+^ without requiring a gating modifier such as riluzole (27). That is, for human *KCNN4* (hKCa3.1), the threshold for current activation is ~100 nM Ca^2+^, the EC_50_ is ~270 nM, and the current is essentially fully activated at 1 μM Ca^2+^ (6, 7).

**Figure 2:**
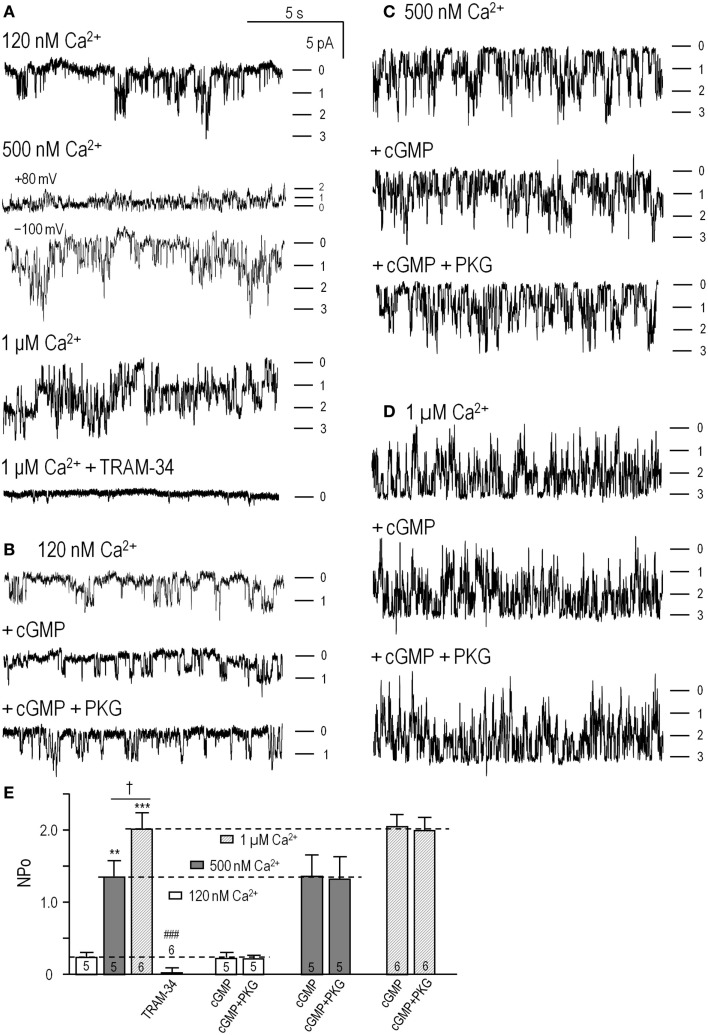
**cGMP-protein kinase (PKG) did not directly affect KCa3.1 channel activity**. Inside-out patches were excised from HEK293 cells that had been transfected with wild-type human *KCNN4* (KCa3.1). The bath and pipette solutions both contained 140 mM potassium, and unless otherwise indicated, inward single-channel currents were recorded at a membrane potential of −100 mV. **(A)** KCa3.1 channel activity was recorded with intracellular solutions containing ATP and 120 nM, 500 nM, or 1 μM free Ca^2+^, sequentially perfused into the bath. At the end of the recording, the KCa3.1 selective blocker, 1 μM TRAM-34, was perfused in. Patches usually contained multiple channels, and the dashes indicate the closed level and opening of 1, 2, or 3 channels. **(B–D)** At each Ca^2+^ concentration (120 nM, 500 nM, 1 μM), the bath was sequentially perfused with cGMP (100 μM), and cGMP + PKG holoenzyme (1 U/μL). **(E)** Summarized data show *NP*_o_ in control bath solution and 4–6 min after adding cGMP or cGMP + PKG. Data are expressed as mean ± SEM for the number of patches indicated on the bars. The dashed lines indicate the *NP*_o_ value in control bath solution at each Ca^2+^ concentration. A two-way ANOVA with Tukey’s *post hoc* test shows that activity increased with intracellular Ca^2+^ (**p < 0.01 for 500 nM Ca^2+^, and ***p < 0.001 for 1 μM Ca^2+^) and was significantly reduced by 1 μM TRAM-34 (only data for 1 μM Ca^2+^ shown; ^###^*p* < 0.001). There were no differences with cGMP or cGMP/PKG treatments.

Inside-out patches were excised into a bath (intracellular) solution containing 100 μM ATP and free Ca^2+^ concentrations of 120 nM, 500 nM, or 1 μM (Figure 2). The intracellular and extracellular solutions contained symmetrical high K^+^ (140 mM) to set the Nernst potential to 0 mV, increase the unitary inward current amplitude, and expose the innate inward rectification of the single-channel current. One to three channels were usually active in each patch, their activity was stable for several minutes, and channel activity was recorded at –100 mV. Channel activity was quantified as *NP*_o_: the number of active channels, *N*, times the open probability, *P*_o_. Using representative 2 min-long segments of each recording, thresholds were set for the closed level and each open level (based on amplitude histograms; see Methods).

As expected for KCa3.1, channel activity (*NP*_o_) increased with increasing free Ca^2+^ (Figures 2A–D; summarized in Figure 2E), and the current was fully blocked by 1 μM TRAM-34 (Figures 2A,E). For the 500 nM Ca^2+^ concentration, both inward (at –100 mV) and outward (at +80 mV) currents are shown to illustrate the inward rectification in symmetrical high K^+^ solutions (~32 pS at –100 mV, ~12 pS at + 80 mV in our recordings) that is characteristic of this channel (3, 4, 7). Overall, the channels were identified as KCa3.1 from their Ca^2+^ dependence, voltage-independent activity (–100 to +80 mV tested), inward rectification, reversal at ~0 mV (not shown), and block by 1 μM TRAM-34. Sequential addition of 100 μM cGMP [required for PKG activation (46)] and the PKG holoenzyme (1 U/μL) did not affect the channel activity or Ca^2+^ dependence (Figures 2B–D). As summarized in Figure 2E, there were no differences in *NP*_o_ values with or without cGMP or cGMP + PKG at any Ca^2+^ concentration. At 120 nM Ca^2+^, *NP*_o_ was 0.23 ± 0.05 in control solution, 0.22 ± 0.06 after adding cGMP, and 0.21 ± 0.04 after adding PKG (*n* = 5). At 500 nM Ca^2+^, *NP*_o_ was 1.18 ± 0.28 in control solution, 1.20 ± 0.32 after adding cGMP, and 1.17 ± 0.36 after adding PKG (*n* = 5). At 1 μM Ca^2+^, *NP*_o_ was 1.89 ± 0.21 in control solution, 2.01 ± 0.23 after adding cGMP, and 2.00 ± 0.24 after adding PKG (*n* = 6).

These results on isolated channels show that cGMP and PKG did not change the number or activity of the channels or their Ca^2+^ dependence, and amplitude histograms (not illustrated) showed that the unitary current amplitude was unaffected. This is in contrast to our recent finding that activated PKA directly reduced P_o_ by ~45%, and required the channel’s PKA phosphorylation site (27). The present results provide strong evidence against direct channel phosphorylation by PKG.

### PKG increases ROS production, which activates KCa3.1 current through a CaMKII-mediated pathway

**Figure 3:**
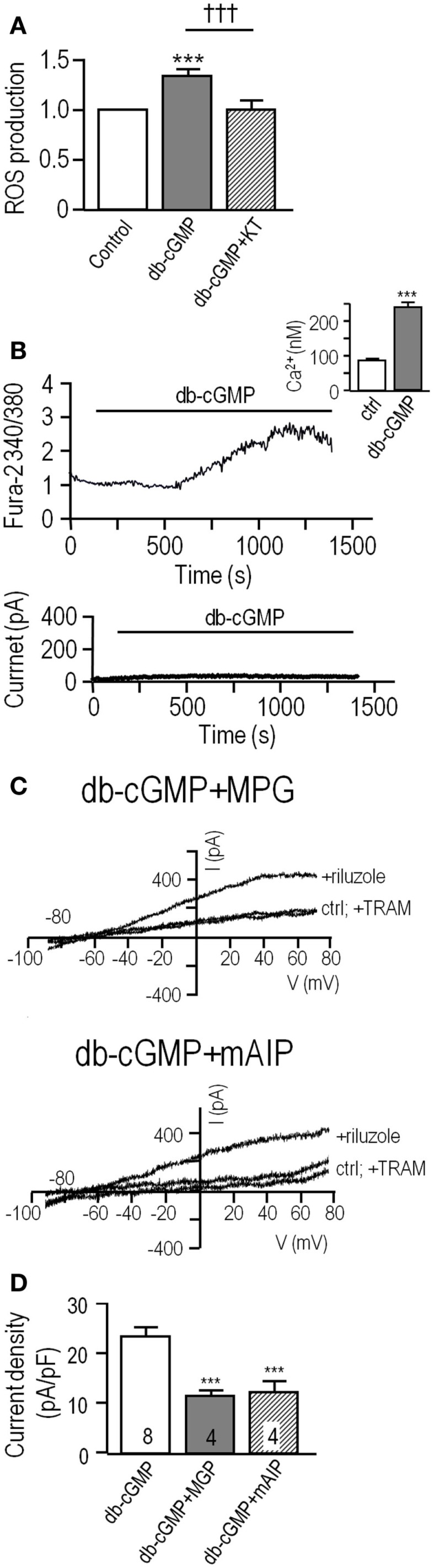
**PKG increases production of reactive oxygen species (ROS), which activates KCa3.1 current through a Ca^2+^ and CaMKII-mediated pathway**. **(A)** Summarized data showing ROS production by unstimulated MLS-9 microglial cells (control), and after treatment (20 min; 37°C) with db-cGMP (100 μM), with or without the PKG inhibitor, KT5823 (1 μM). Values are expressed as mean ± SEM (*n* = 5), and compared using a one-way ANOVA with Tukey’s *post hoc* test. ***p < 0.001 treatment versus control; †††*p* < 0.001 with and without PKG inhibitor. **(B)** Acute application of db-cGMP increases intracellular Ca^2+^ in MLS-9 cells but does not activate KCa3.1 current. *Upper panel*: Representative Fura-2 recording, in which 100 μM db-cGMP was bath applied during the period marked by the horizontal bar. The inset shows calibrated free intracellular Ca^2+^ concentration as mean ± SEM, *n* = 19 cells (***p < 0.001, Student’s *t*-test). *Lower panel*: Representative time course of current in a perforated-patch recording (same solutions and voltage protocols as Figure 1) in which db-cGMP (100 μM) was bath applied as indicated by the horizontal bar. **(C)** KCa3.1 current potentiation by db-cGMP is prevented by the ROS scavenger, MPG, and the CaMKII inhibitor, mAIP. Each set of three traces shows representative currents before and after adding 300 μM riluzole, with or without 1 μM TRAM-34. *Upper panel*: Representative recording from an MLS-9 cell pre-treated with 100 μM db-cGMP and the ROS scavenger, MPG (500 μM), for 20 min at room temperature. *Lower panel*: Cells were pre-treated with 100 μM db-cGMP and the CaM kinase II inhibitor, mAIP (1 μM) for 20 min at room temperature. **(D)** Summarized data from a population study with treatments as in panel C. The TRAM-34-sensitive KCa3.1 current is expressed as mean ± SEM for the number of cells indicated on each bar, and was compared using a one-way ANOVA with Tukey’s *post hoc* test; ***p < 0.001.

The lack of effect of cGMP and PKG on excised patches (Figure 2) suggested that intracellular signaling was required for the current enhancement in MLS-9 cells (Figure 1). Therefore, we used perforated-patch recordings to maintain intracellular signaling when conducting experiments on native channels in MLS-9 cells and primary microglia. We first considered ROS because PKG increases ROS production in cardiac cells and neurons (28–30). Treating MLS-9 cells with db-cGMP increased ROS production by 38 ± 5% (Figure 3A; *n* = 5; *p* < 0.001), an effect that was prevented by the PKG inhibitor, KT5823. ROS can activate CaMKII (21, 47) and, as described in the Introduction, CaMK regulates native KCa3.1 channels in T lymphocytes (8). Intriguingly, CaMKII can be activated by ROS without elevated intracellular Ca^2+^ (21) or in a long-lasting Ca^2+^-independent manner after a transient Ca^2+^ elevation (47).

Acute application of db-cGMP to MLS-9 cells evoked a modest rise in intracellular Ca^2+^, which peaked at ~15 min and began to decline despite the continued presence of db-cGMP (Figure 3B). The calibrated Fura-2 signal indicates that basal Ca^2+^ was 88 ± 4 nM (*n* = 37) [consistent with our earlier studies (23, 31)], and the peak after applying db-cGMP was 242 ± 12 nM (*n* = 19), which was not sufficient to activate the KCa3.1 current (*n* = 6 cells tested; example in Figure 3B). In a separate experiment (Figure 3C), MLS-9 cells were treated with db-cGMP to increase the KCa3.1 current (activated by riluzole, as in Figure 1), and simultaneously with a membrane-permeant CaMKII inhibitor (1 μM; myristolated autocamtide-2 related inhibitory peptide; mAIP) or a ROS scavenger (500 μM; MPG). MPG is a synthetic oxyradical scavenger that is effective for superoxide (O_2_^−^), hydrogen peroxide (H_2_O_2_), and hydroxyl radical (OH^−^) (48). Effects of MPG directly implicate ROS, which is important because the probe used to measure ROS production (CM-H_2_DCFDA) can also detect peroxynitrite (42). As summarized in Figure 1D, the current density was 23.4 ± 0.9 pA/pF (*n* = 8) after db-cGMP addition alone, and reduced by MPG to 11.1 ± 0.8 pA/pF (*n* = 4; *p* < 0.001) and by mAIP to 11.5 ± 2.2 pA/pF (*n* = 4; *p* < 0.001). Together, these data provide evidence that the enhancement in KCa3.1 currents by PKG requires both ROS and CaMKII.

### Application of H_2_O_2_ increases KCa3.1 current through a CaMKII-dependent pathway, not through direct activation of the channel

**Figure 4:**
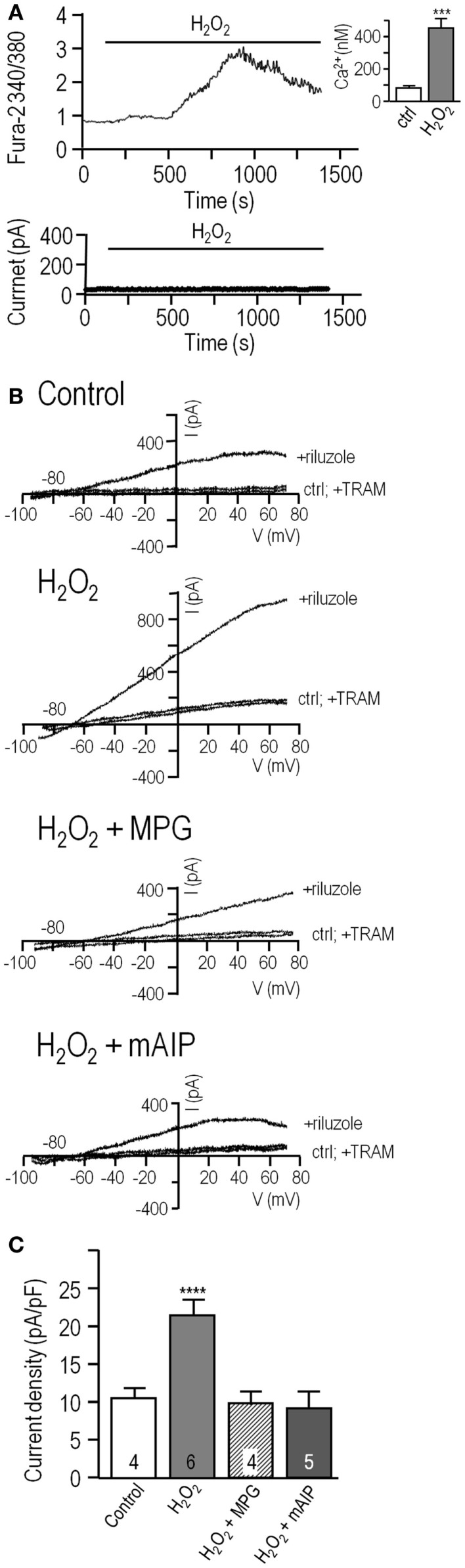
**Direct application of H_2_O_2_ increases the KCa3.1 current through a CaMKII-mediated pathway**. **(A)** Hydrogen peroxide elevates intracellular Ca^2+^ but does not directly activate KCa3.1 current in MLS-9 cells. *Upper panel*: Representative Fura-2 recording, in which 1 mM H_2_O_2_ was bath applied during the period marked by the horizontal bar. The inset shows calibrated free intracellular Ca^2+^ concentration as mean ± SEM, *n* = 18 cells (***p < 0.001, Student’s *t*-test). *Lower panel*: Representative current in a perforated-patch recording (same solutions and voltage protocols as Figure 1) with 1 mM H_2_O_2_ bath applied as indicated. **(B)** Representative KCa3.1 current traces in perforated-patch recordings, representative currents before and after adding 300 μM riluzole, with or without 1 μM TRAM-34. From top to bottom: control cell, cell pre-treated with 1 mM H_2_O_2_ for 10 min at room temperature, cell pre-treated with both 1 mM H_2_O_2_ and the ROS scavenger, MPG (500 μM; 10 min, room temperature), cell pre-treated with 1 mM H_2_O_2_, and the CaMKII inhibitor, mAIP (1 μM; 10 min, room temperature). **(C)** Summarized data from a population study with treatments as in panel **(B)**. The TRAM-34-sensitive KCa3.1 current is expressed as mean ± SEM for the number of cells indicated on each bar and was compared using a one-way ANOVA with Tukey’s *post hoc* test; ****p < 0.0001.

To further analyze the ROS-mediated increase in KCa3.1 current, we tested acute application of the relatively stable hydrogen peroxide (1 mM H_2_O_2_) molecule. Treating MLS-9 cells with H_2_O_2_ evoked a moderate, transient rise in intracellular Ca^2+^ (Figure 4A) that peaked at 456 ± 33 nM (*n* = 18) by 11.6 ± 0.1 min after treatment. The peak elevation in Ca^2+^ evoked by H_2_O_2_ was higher than for db-cGMP and occurred ~5 min sooner. Again, it was insufficient to activate the KCa3.1 current in MLS-9 cells (*n* = 6 cells tested; example in Figure 4A), which requires supra-micromolar concentrations (described above). In contrast, in perforated-patch recordings from cells that were pre-incubated with 1 mM H_2_O_2_, the KCa3.1 current was more than twofold larger (21.5 ± 1.6 pA/pF; *n* = 6; *p* < 0.0001) than in control cells (10.3 ± 0.5 pA/pF; *n* = 4; Figures 4B,C). The potentiation of the current was prevented if cells were simultaneously pre-treated with the ROS scavenger (500 μM MGP) or the CaMKII inhibitor (1 μM mAIP). The current density remained at 9.5 ± 1.4 pA/pF in MGP-treated cells and 9.2 ± 1.8 pA/pF in mAIP-treated cells. These data show that KCa3.1 function can be enhanced by this identified, stable ROS species through a similar pathway involving CaMKII.

**Figure 5:**
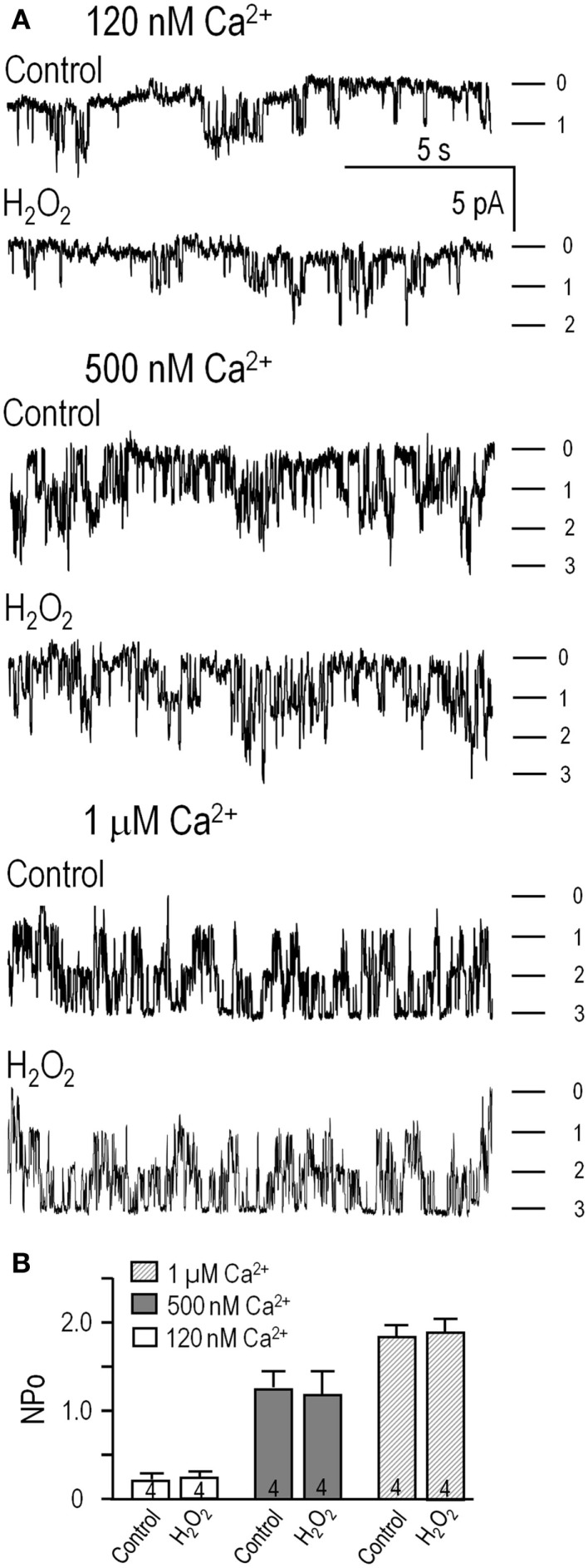
**H_2_O_2_ did not directly affect the KCa3.1 channels**. **(A)** HEK293 cells transfected with hKCa3.1 were used to assess channel activity in inside-out patches in intracellular (bath) solutions containing 120 nM, 500 nM, or 1 μM free Ca^2+^ (as in Figure 2), with or without perfusing in 1 mM H_2_O_2_. Inward single-channel currents were recorded at −100 mV. **(B)** Summarized data show *NP*_o_ in control bath solution, and 4–6 min after adding H_2_O_2_. Data are expressed as mean ± SEM from four patches per Ca^2+^ concentration, and a one-way ANOVA with Tukey’s *post hoc* test showed no differences following treatments.

Next, inside-out patches from transfected HEK293 cells were exploited to ask whether H_2_O_2_ can directly affect channel activity (*NP*_o_; calculated as in Figure 2). There were 1–3 active channels in each patch, channel activity increased with increasing Ca^2+^ (Figure 5), and the *NP*_o_ values were the same as in Figure 2. Perfusing 1 mM H_2_O_2_ into the bath did not affect channel activity at any of the Ca^2+^ concentrations (Figure 5A, summarized in Figure 5B). At 120 nM Ca^2+^, *NP*_o_ was 0.21 ± 0.05 (*n* = 4) and 0.23 ± 0.04 after adding H_2_O_2_. In 500 nM Ca^2+^, *NP*_o_ was 1.17 ± 0.19 (*n* = 4) and 1.09 ± 0.21 after adding H_2_O_2_. At 1 μM Ca^2+^, *NP*_o_ was 1.79 ± 0.26 (*n* = 4) and 1.88 ± 0.31 after adding H_2_O_2_. Thus, treatment with this ROS did not directly affect KCa3.1 activity, number of active channels, or their Ca^2+^ sensitivity; nor was the amplitude of single-channel currents affected (amplitude histograms; not shown). This supports the view that, in order to exert their modulatory effects on KCa3.1 channels, H_2_O_2_ and CaMKII require intact cells and possibly another mediator.

### In alternative-activated rat microglia, increased ROS production potentiates the KCa3.1 current through a PKG- and CaMKII-dependent pathway

**Figure 6:**
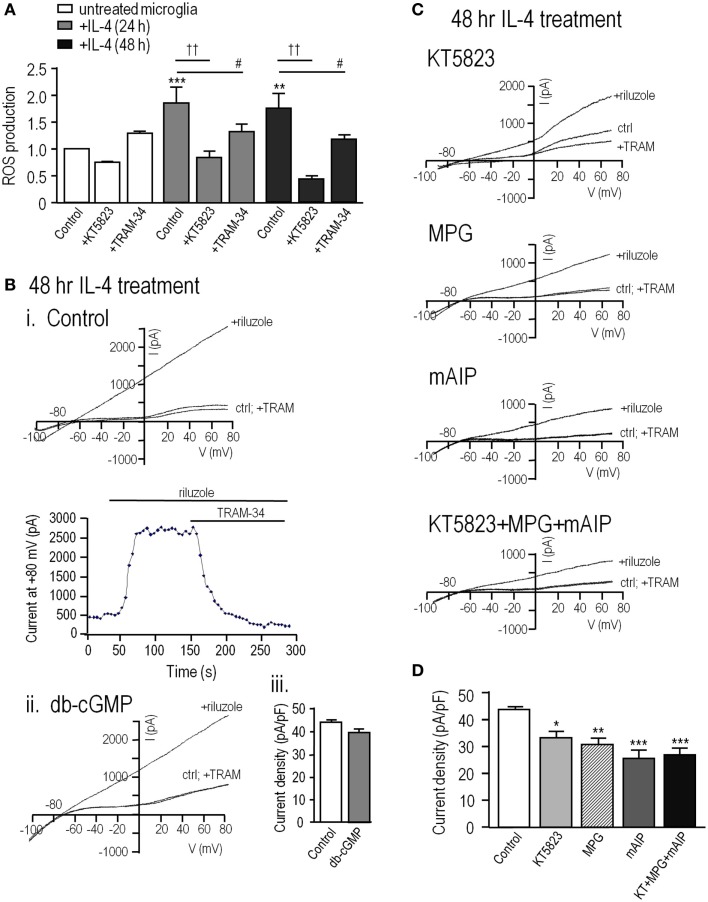
**In alternative-activated rat microglia, ROS production requires and potentiates KCa3.1 channel activity through the ROS-PKG-CaMKII pathway**. To evoke alternative activation, primary rat microglia were treated with rat recombinant interleukin-4 (IL-4); 20 ng/mL for 24 or 48 h. **(A)** Summarized data show ROS production, detected by CM-H_2_DCFDA (see Methods) in untreated versus IL-4 treated microglia. Under each condition, separate batches of microglia were exposed to the PKG inhibitor (1 μM KT5823) or the KCa3.1 blocker (1 μM TRAM-34) for 24 or 48 h at 37°C. Values are expressed as mean ± SEM (*n* = 6 replicates experiments each), and compared using a two-way ANOVA with Tukey’s *post hoc* test. **p < 0.01 and ***p < 0.001 for non-activated versus IL-4 treated cells; ^#^*p* < 0.05 and ††*p* < 0.01, for drug treatments, as indicated. **(B)** KCa3.1 currents were recorded in alternative-activated microglia 48 h after IL-4 treatment, using the perforated-patch configuration and the same solutions and voltage protocols as in Figure 1. (i) Representative currents from a cell before and after adding the KCa3.1 activator, 300 μM riluzole, and the KCa3.1 blocker, 1 μM TRAM-34. (ii) A cell pre-treated with 100 μM db-cGMP for 20 min at room temperature. (iii) Summarized data from a population study, in which the TRAM-34-sensitive KCa3.1 current amplitude is expressed as mean ± SEM (*n* = 4 cells each). The difference was non-significant based on Student’s *t*-test. **(C)**. KCa3.1 currents were recorded from alternative-activated microglia, with and without the activator, riluzole, as in panel B. All drug pre-treatments were for 1 h at 37°C. From top to bottom, different microglia were treated with 1 μM KT5823; the ROS scavenger, 500 μM MPG; the CaMKII inhibitor, 1 μM mAIP; KT5823, MPG and mAIP. **(D)**. Summarized data from a population study of experiments as in panel **(C)**. The TRAM-34-sensitive KCa3.1 current is expressed as the mean ± SEM (*n* = 5 cells each), and was compared using a one-way ANOVA with Tukey’s *post hoc* test; *p < 0.05, **p < 0.01, ***p < 0.001.

The above results on MLS-9 cells show that ROS and cGMP increase KCa3.1 current through a pathway requiring PKG and CaMKII. It was important to determine whether the same pathway regulates KCa3.1 in primary cultured microglia. For these experiments, we used alternative-activated (IL-4 treated) rat microglia, which have upregulated *KCNN4* expression and a much larger KCa3.1 current than resting microglia (20), and in which KCa3.1 contributes to Ca^2+^ signaling (27) and migration (20). Here, we found that at 24 and 48 h after IL-4 treatment, ROS production was increased by 93 ± 31% (*p* < 0.01) and 78 ± 21% (*p* < 0.01), respectively (Figure 6A). The PKG inhibitor, KT5823, decreased this induced ROS production by 53% at 24 h and 73% at 48 h after IL-4 treatment, but did not significantly affect resting microglia. The enhanced ROS production in alternative-activated microglia was moderately dependent on KCa3.1 channels, as TRAM-34 reduced it by 29% at 24 h and 31% at 48 h after IL-4 treatment.

Perforated-patch recordings from alternative-activated rat microglia were used to assess KCa3.1 regulation. For all recordings, the bath contained 100 nM apamin to block the KCa2.3 channels (41), KCa3.1 was activated by riluzole, and 1 μM TRAM-34 was added at the end of each recording to quantify the TRAM-34-sensitive KCa3.1 component. This subtraction procedure was used because primary rat microglia have a voltage-dependent Kv1.3 current, which can be seen in control traces before riluzole was added to activate KCa3.1. Note, however, that riluzole reduces the microglial Kv1.3 current (20). As for MLS-9 cells, riluzole-activated a KCa3.1 current in every microglia cell that was examined. Again, the current activation was not voltage-dependent; it reversed near the K^+^ Nernst potential, and was fully blocked by 1 μM TRAM-34 (Figure 6B). The only differences from MLS-9 cells were that the current was several-fold larger in IL-4-treated primary microglia (compare with Figure 1) and pre-treatment with db-cGMP did not further increase it. The current density was 43.7 ± 1.4 pA/pF in control microglia and 39.9 ± 1.9 pA/pF in cells pre-treated with db-cGMP, which raises the possibility that the current is already maximal in alternative-activated microglia. The key finding was that the KCa3.1 current in primary microglia was also regulated by ROS, PKG and CaMKII, as it was in MLS-9 cells. The current density was reduced from 43.7 ± 1.4 pA/pF in control microglia to 29.8 ± 2.1 pA/pF by the ROS scavenger (MPG), to 32.6 ± 2.2 pA/pF by the PKG inhibitor (KT5823), and to 23.8 ± 2.7 pA/pF by the CaMKII inhibitor (mAIP) (Figures 6C,D). Evidence for a common regulatory pathway was that combining all three inhibitors did not further reduce the current, as it would have for separate, additive pathways. Together, these results suggest that in alternative-activated microglia, the KCa3.1 current is maximally activated through a regulation pathway involving endogenous PKG, ROS, and CaMKII.

## Discussion

**Figure 7:**
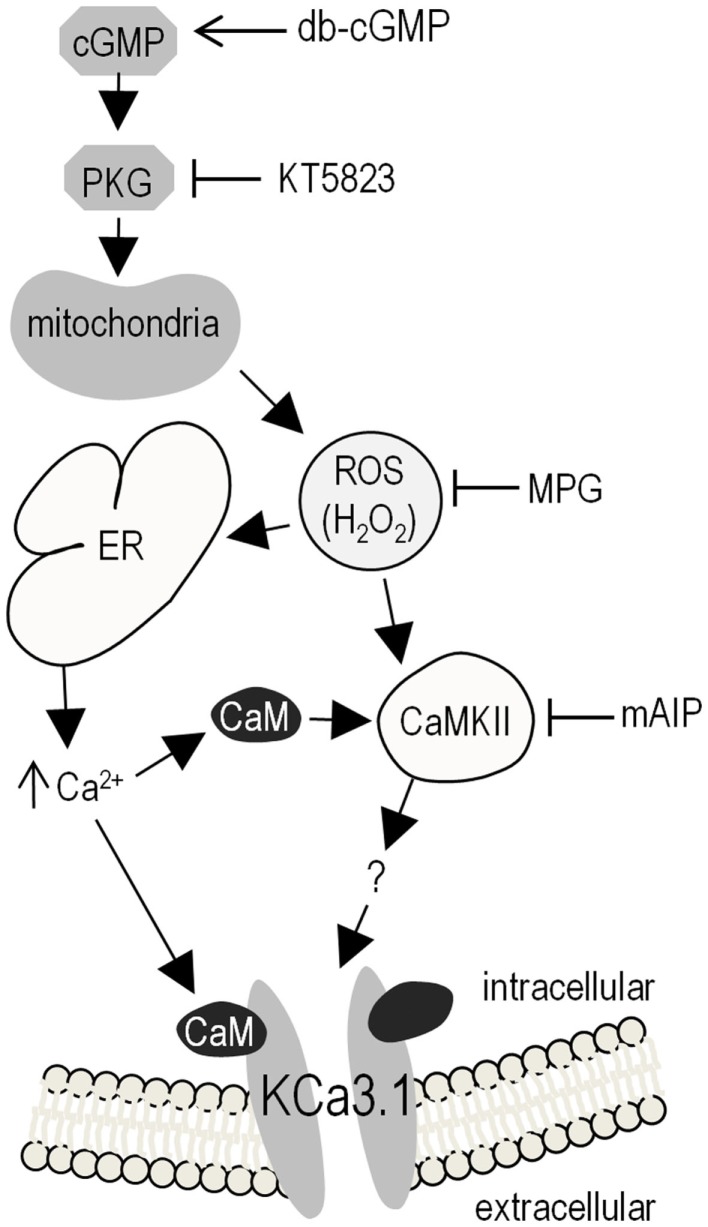
**Summary of results and proposed model of KCa3.1 regulation in microglia**. Elevating intracellular cGMP activates PKG, which can then phosphorylate numerous downstream cellular targets, one of which triggers mitochondrial production of ROS (proposed to be via the “5-hydroxydecanoate-sensitive factor” that is likely the mitoK_ATP_ channel). Intracellular ROS can then contributes to KCa3.1 regulation through its role as a signaling intermediate; e.g., by evoking Ca^2+^ release from intracellular stores on the ER, leading to CaM-dependent activation of CaMKII, which then increases KCa3.1 activity (by an unknown mechanism). *Activator used*: 100 μM db-cGMP (membrane-permeant cGMP analog) to activate PKG. *Inhibitors used*: 1 μM KT5823 for PKG; 500 μM MPG as a general ROS scavenger (including O_2_^–^, H_2_O_2_, OH^∙^); 1 μM mAIP for CaMKII. *Acronyms*: CaMKII, Ca^2+^/CaM-dependent protein kinase II; cGMP, cyclic guanosine monophosphate; db-cGMP, dibutyryl-cyclic guanosine monophosphate; ER, endoplasmic reticulum; H_2_O_2_, hydrogen peroxide; mAIP, myristolated autocamtide-2 related inhibitory peptide; mitoK_ATP_, mitochondrial ATP-sensitive potassium channel; MPG, *N*-(2-mercaptopropionyl)glycine; O_2_^–^, superoxide; OH^∙^, hydroxyl radical; PKG, cGMP-dependent protein kinase; ROS, reactive oxygen species.

Figure 7 summarizes our results and presents a model of KCa3.1 post-translational regulation based on our observations and the literature. Elevating cGMP activates PKG, which increases ROS production, evokes Ca^2+^ release from intracellular stores, which binds to CaM, and opens the KCa3.1 channel. CaM also activates CaMKII, which enhances the KCa3.1 current through an unknown mechanism. This is the first report of KCa3.1 regulation by cGMP/PKG and ROS. In comparing the present results with the literature, it is important to note several experimental considerations.

One major consideration is the patch-clamp configuration. When studying signaling pathways that regulate ion channel function, diffusible regulators can be lost or compromised in whole-cell recordings or excised patches. Therefore, we performed all recordings of native channels in the perforated-patch configuration to maintain the cytoplasmic integrity, allow intracellular Ca^2+^ to “free-run,” and prevent loss of soluble mediators. We applied all modulators by pre-incubation (10–60 min) before recordings were established; i.e., db-cGMP with or without KT5823 (PKG inhibitor), MPG (ROS scavenger), or mAIP (CaMKII inhibitor); H_2_O_2_ with or without MPG or mAIP; or in alternative-activated (IL-4 treated) primary microglia with or without KT5823, MPG or mAIP. Results can differ in excised patches (e.g., our observed lack of effect of PKG in inside-out patches) or in whole-cell recordings. For instance, we showed that cAMP/PKA increased activity of Kv1.3 channels in intact human T lymphocytes but this regulation was lost in whole-cell recordings (49). In a pilot study using whole-cell recordings from MLS-9 cells, acute application of db-cGMP (data not shown) did not increase the KCa3.1 current, and this provided the first evidence for loss of a soluble mediator.

Another concern is whether the channels have been convincingly identified as KCa3.1. Following cloning of *KCNN4* [the gene coding for KCa3.1 (5–7)], KCa3.1 currents were identified by their requirement for Ca^2+^ (EC_50_ 200–700 nM); voltage-independent gating; reversal near the Nernst potential for K^+^ (–85 mV with physiological internal and external K^+^), an inward-rectifying single-channel conductance in symmetrical high K^+^ solutions (~10 pS at positive potentials and 25–35 pS at very negative potentials); block by TRAM-34 (selective at ≤1 μM) and the less selective blockers, charybdotoxin (ChTx; IC_50_ ~5 nM), and clotrimazole (IC_50_ < 70 nM). Here, and in several recent papers on microglia, MLS-9 cells (20, 27, 31), we identified the KCa3.1 current by several of these criteria: (i) voltage-independent gating and thus, current seen at all voltages tested; (ii) a need for elevated intracellular Ca^2+^ (although unusually high); (iii) current enhancement by positive gating modulators (riluzole, 1-EBIO, NS309); and (iv) essentially full block by 1 μM TRAM-34.

Four papers have implicated cGMP and/or PKG in activating Ca^2+^-dependent K^+^ channels; two published before the channel was cloned and two afterward. In the early study of vascular smooth muscle cells (50), the type of K^+^ channel activated by cGMP was not identified, but is unlikely to be KCa3.1. That is, the whole-cell current was depolarization-activated and blocked by 10 mM tetraethylammonium (TEA) as well as 200 nM ChTx, which is more consistent with large-conductance Ca^2+^-dependent K^+^ (BK) channels. It is now well known that BK channels are activated by the NO-cGMP-PKG pathway [reviewed in Ref. (51–53)]. In the study on rat cortical collecting duct epithelial cells, inside-out patches showed that two channel types (28 and 85 pS) were activated by cGMP and this was prevented by the PKG inhibitor, KT5823 (54). However, these recordings were made in the absence of intracellular Ca^2+^ or a gating modifier, and thus neither is likely to be KCa3.1. The two later papers reported that cGMP regulates a clotrimazole-sensitive channel, and one concluded that it was KCa3.1. We reported that the IC_50_ for KCa3.1 block by clotrimazole was 40 and 56 nM for native channels in human T lymphoblasts and hKCa3.1 expressed in CHO cells, respectively (8) and we subsequently used 200 nM in functional studies (14). However, clotrimazole also inhibits Ca^2+^ channels (55), and at micromolar concentrations, it inhibits NMDA channels (56), TRPM2 channels (57), and several K^+^ channels; i.e., transient outward, ultra-rapid delayed-rectifier, hERG and KCNQ1/KCNE1 channels (58). The third paper on potential KCa channel regulation by PKG showed that a K^+^ current in interstitial cells of Cajal was activated by Ca^2+^ and by the nitric oxide donor, sodium nitroprusside (SNP), blocked by 1 μM clotrimazole, and had a single-channel conductance of ~38 pS (59), which are consistent with KCa3.1. However, it exhibited a steeply voltage-dependent activation at –40 mV, which is not consistent with KCa3.1. Finally, a study of single-channel activity in human dermal fibroblasts showed that activity was increased by the NO donor, S-nitroso-*N*-acetylpenicillamine (SNAP) in cell-attached patches, and by applying cGMP + PKG to the cytoplasmic face of inside-out patches (60). The channel was blocked by 10 μM clotrimazole, was depolarization activated at about –40 mV, and had a large single-channel conductance (116 pS reported, >150 pS in some recordings); properties that are inconsistent with KCa3.1.

We began this study by noting that KCa3.1 has a single consensus motif for phosphorylation by PKG (^331^RKES^334^ in the human gene), but our results rule out this mechanism. First, applying PKG (with cGMP and ATP) to the cytoplasmic membrane face had no effect on channel activity and second, when S334 is phosphorylated by PKA the current is decreased, not increased (27). Failure of a kinase to phosphorylate a potential consensus motif is not unusual. For instance, PKG might have a low affinity due to conformational considerations; activated PKG (2 PKG monomers + 4 cGMP molecules) is ~148 kDa compared with the catalytic subunit of PKA (43.5 kDa). Our results instead support a linear mechanism for enhancing the native KCa3.1 current: elevating cGMP (by adding membrane-permeant db-cGMP) activates PKG (inhibited by KT5823), evokes ROS production (mimicked by H_2_O_2_ and inhibited by MPG), elevates intracellular Ca^2+^ (but not high enough to directly activate the channel), and activates CaMKII (inhibited by mAIP). Activation of KCa3.1 by CaMKII is consistent with our earlier study in which this current in human T lymphoblasts was inhibited by the CaMK antagonist, KN-62 (8).

Several aspects would be worth considering in future studies. (i) We do not know the mechanism by which CaMKII increases the KCa3.1 current. One possibility is that it promotes channel trafficking/insertion into the surface membrane or, conversely, reduces endocytosis. CaM is involved in assembly and trafficking of KCa3.1 (26) but there is no evidence that CaMKII is involved. In expression systems, KCa3.1 turns over rapidly through clathrin-mediated endocytosis and is then targeting for lysosomal degradation (61). (ii) CaMK-dependent changes in gene transcription can produce long-lasting cellular outcomes, such as learning and memory [reviewed in Ref. (62, 63)] but we think altered gene expression is unlikely. For instance, the CaMKII inhibitor, mAIP, was only applied for 1 h to alternative-activated microglia. (iii) In alternative-activated microglia, ROS production was increased, and dependent on both PKG signaling and KCa3.1 channels. We previously found that KCa3.1 was necessary for efficient ROS production in classical-activated microglia. Because ROS increased the KCa3.1 current this might be a positive feedback mechanism to increase KCa3.1 contributions to microglia functions under conditions of oxidative stress; e.g., after acute injuries, such as stroke. (iv) When exposed to classical-activation stimuli (e.g., LPS) or in conjunction with phagocytosis, microglia produce ROS through an NADPH oxidase (NOX)-mediated respiratory burst (64, 65). While ROS play anti-microbial roles, endogenously generated ROS can potentially feed back onto physiological functions of microglia; e.g., activating NFκB and synthesis of TNFα (66). In contrast, ROS can reduce classical activation of peritoneal macrophages (67), suggesting complex roles in regulating immune cell functions. One question is whether the source matters; i.e., ROS produced by NOX versus mitochondria. We think the long-lasting ROS production in IL-4-treated microglia (elevated for at least 2 days) is likely mediated by mitochondria, because PKG is known to stimulate mitochondrial ROS production in cardiomyocytes and a neuroblastoma cell line (28–30). However, the IL-4 signaling pathway increased ROS production through PI3K-dependent activation of NOX enzymes in an epithelial cell line (68); thus, we cannot rule out NOX contributions.

### Broader implications

KCa3.1 is expressed in numerous cell types, including red blood cells, some immune cells [see Introduction, and reviewed in Ref. (69)], neurons (15–17), epithelia (70), vascular smooth muscle and endothelial cells (71, 72), fibroblasts (73, 74), stem cells (75, 76), and cancer cells (77, 78). Because KCa3.1 plays diverse roles in these cells (e.g., proliferation, volume regulation, migration, cytokine production, and others) its post-translational regulation by cGMP/PKG, ROS, and Ca^2+^/CaM/CaMKII is likely to have broad consequences. There are many opportunities for KCa3.1 to be regulated by cGMP/PKG signaling because this pathway can be activated through NO produced by nNOS (neurons), eNOS (endothelial cells) and iNOS (innate immune cells), and acting on soluble guanylate cyclase. In microglia, iNOS is upregulated and NO is produced in the classical-activated state. Pro-inflammatory mediators are induced by LPS, and there is evidence that atrial natriuretic peptide (ANP)-induced cGMP/PKG signaling can reduce this (79). In microglia, the roles of PKG are not well known but we found that PKG is involved in ROS production in alternative-activated microglia. Further evidence for involvement of PKG and ROS in alternative activation is that PKG increased IL-4 production by Th2 lymphocytes (80), and ROS increased IL-4 release by macrophages (81, 82). The widespread coincidence of this channel and signaling pathway is reinforces the importance of future studies addressing its regulation in other cell types.

## Author Contributions

LS, RF, and RW contributed to the conception, design of this study, and wrote the manuscript. RF and RW performed the experiments. LS, RF, and RW agree to be accountable for all aspects of the work.

## Conflict of Interest Statement

The authors declare that the research was conducted in the absence of any commercial or financial relationships that could be construed as a potential conflict of interest.
